# Praziquantel and factor H recruitment differentially affect the susceptibility of *Schistosoma mansoni* to complement-mediated damage

**DOI:** 10.3389/fimmu.2024.1474358

**Published:** 2024-11-12

**Authors:** Anna E. van Beek, Hannah Jeanguenat, Cécile Häberli, Richard B. Pouw, Christina Lamers, Gábor Pál, Péter Gál, Christoph Q. Schmidt, Daniel Ricklin, Jennifer Keiser

**Affiliations:** ^1^ Department of Medical Parasitology and Infection Biology, Swiss Tropical and Public Health Institute, Allschwil, Switzerland; ^2^ University of Basel, Basel, Switzerland; ^3^ Department of Pharmaceutical Sciences, University of Basel, Basel, Switzerland; ^4^ Sanquin Research and Landsteiner Laboratory of the Amsterdam University Medical Centers (UMC), University of Amsterdam, Amsterdam, Netherlands; ^5^ Department of Biochemistry, Eötvös Loránd University, Budapest, Hungary; ^6^ Institute of Molecular Life Sciences, HUN-REN Research Centre for Natural Sciences, Hungarian Research Network, Budapest, Hungary; ^7^ Institute of Experimental and Clinical Pharmacology, Toxicology and Pharmacology of Natural Products, University of Ulm Medical Center, Ulm, Germany

**Keywords:** complement, *Schistosoma mansoni*, praziquantel, complement factor H, complement evasion, host-pathogen interactions

## Abstract

**Background:**

Schistosomes are highly efficient evaders of human immunity, as evident by their ability to survive in human blood for years. How they protect themselves against the constant attack by a key element of innate immunity, the complement system, has remained unclear. In this study, new light is shed on the interaction between distinct life-cycle stages of *Schistosoma mansoni* and the human complement system.

**Results:**

We demonstrate that schistosomula, the young stage assumed immediately after cercaria penetration of the skin, are extremely vulnerable towards complement-mediated killing as only 10-20% survive. The survival rate increases to 70% already within 30 minutes and reaches close to 100% within two hours. Pathway-specific complement inhibitors revealed the alternative pathway of complement activation as the main contributor to killing and damage of the schistosomula. Moreover, the complement regulator factor H is recruited by the schistosomula in this early stage to evade killing. Surviving parasites appear fully viable despite the ongoing complement attack, as demonstrated by the deposition of C3 fragments. However, when exposed to the widely used schistocidal drug praziquantel, the vulnerability of 24 h-old schistosomula towards complement-mediated killing is notably increased; no such effect was observed for mefloquine or oxamniquine. Similar to the younger life-cycle stages, adult worms remain under complement attack. C3 fragments were found all over the outer surface (tegument), deposited mostly on the ridges and not on the tubercles.

**Conclusion:**

The recruitment of factor H merits more detailed studies that pinpoint the molecules involved and elucidate the novel possibilities to intercept the uncovered immune evasion therapeutically. That praziquantel and complement work in synergy is surprising and may in the future result in enhanced understanding of the drug’s mechanism of action.

## 1 Introduction

Schistosomiasis is caused by the helminth species *Schistosoma haematobium*, *S. japonicum* and *S. mansoni* and ranks among the leading neglected tropical diseases in terms of disability-adjusted life years, with roughly 252 million people infected worldwide (1). Control of schistosomiasis entirely relies on a single drug, praziquantel (PZQ), which has been widely used over the past 50 years (2, 3). Despite its therapeutic success, a major limitation of PZQ is that it only kills adult worms, thereby leaving patients vulnerable for re-infection and requiring retreatment to target the initially immature forms after they had matured into drug-susceptible adult worms (4). Schistosomiasis has a profound social and economic impact: children with schistosomiasis suffer from systemic morbidities including anemia, malnutrition and impaired childhood development (1), become more susceptible towards co-infections with malaria (5, 6), and show decreased responses to vaccines [reviewed in (7)].

Human schistosomes undergo an indirect lifecycle alternating between snails as their obligate intermediate host and humans as definitive vertebrate host. From the moment that infective cercariae come into contact with human skin up until their final destination in the mesenteric veins as adult worms, schistosomes require efficient strategies to survive the continuous immunological attack (8, 9). Being a key element of innate immunity and a major initiator of adaptive immune responses, the complement system is at the forefront of this attack (10, 11).

The complement cascade can be initiated by antibodies recognizing their cognate antigen, referred to as the classical pathway (CP), soluble lectins recognizing microbial carbohydrate signatures, defining the lectin pathway (LP), or via engagement of the alternative pathway (AP) on non-self or unprotected self surfaces. Independent of the initiation route, complement activation results in three main effector mechanisms (1): release of anaphylatoxins C3a and C5a, which recruit and activate immune cells (2); deposition of C3b on the target surface, a process referred to as opsonization that facilitates phagocytosis, and (3) formation of membrane-attack complexes (C5b-9, MAC) to cause cell damage or even lysis. Although schistosomes are exposed to this extensive and powerful effector system, they seem to be able to evade complement-mediated destruction once they enter their human host and remain complement-resistant for many years (12, 13).

While there is consensus that lung-stage schistosomula, juvenile and adult worms are resistant towards complement-mediated killing, controversy remains on the vulnerability of cercariae and early skin-stage schistosomula. These stages likely suffer from complement attack owing to the local production of complement factors in the skin (14–17) and diffusion of complement factors from blood. In the past, several studies have reported on the consumption or conversion of human complement components by or deposition of complement fragments on *Schistosoma* (18–27). Whereas some studies demonstrated an almost complete killing of newly transformed schistosomula (NTS) in serum, others could only induce killing in the presence of phagocytes (19, 20, 25, 28). A direct comparison and assessment of divergent study results is often limited by a shortage of experimental details, *e.g.* regarding the number of parasites or the handling of schistosomula before the start of an experiment.

Since these early studies, many technical aspects have improved to investigate the interaction between complement and schistosomes. In our study, we provide new insight into this interaction, demonstrating which complement pathways are involved in the attack and how quickly the schistosomes mount their complement evasion response, which is at least partially mediated by recruitment of the complement regulator factor H (FH). We show that the surviving parasites, both as schistosomula and adult worms, remain under constant complement attack as visualized by the deposition of C3 fragments. Intriguingly, treatment with PZQ appears to counter this resistance by increasing the vulnerability of schistosomes for complement-mediated killing.

## 2 Materials and methods

### 2.1 Ethics statement

All animal experiments were authorized by the regional veterinary office (Canton Basel-Stadt; authorization No. 2070) based on Swiss national and communal regulations.

### 2.2 Proteins and reagents

NTS-medium was prepared by supplementing Medium 199 (Gibco, Waltham MA, USA) with 1% (v/v) penicillin (10,000 U/ml) and streptomycin (10 mg/mL) solution (pen/strep, Bioconcept AG, Allschwil, Switzerland), 1% (v/v) antibacterial/antifungal mix (45% kanamycin, 27% penicillin G, 5-fluorocytosine, 5% chloramphenicol) and 5% (v/v) heat-inactivated fetal calf serum (Bioconcept). Normal human serum (NHS) was obtained from the Basel Blood Donation Centre (Basel, Switzerland), by pooling serum from three anonymous healthy donors (AB+) and storing aliquots at -80°C until use. Heat-inactivated NHS (HI-NHS) was obtained by exposing NHS to 56°C for 1 h. Physostigmine salicylate (PS, cat. no. P1600000, also known as eserine), praziquantel (cat. no. P4668-5G), oxamniquine (cat. no. PZ0393-5MG) and mefloquine (cat. no. M2319-100MG) were purchased from Sigma-Aldrich, Buchs, Switzerland.

Recombinant minibody constructs, consisting of short-chain variable fragments (scFv) fused to a human IgG1 Fc domain, were used as surrogates of the therapeutic antibodies lampalizumab (anti-FD; AP inhibitor), sutimlimab (anti-C1s; CP inhibitor) and eculizumab (anti-C5; terminal pathway inhibitor). Sequences of the three complement-inhibiting minibodies were obtained from the publicly accessible ABCD database (ABCD_AH803, ABCD_AA662 and ABCD_AA552, respectively; https://web.expasy.org/abcd/). The minibodies were produced in HEK293 cells at the Geneva Antibody Facility (https://www.unige.ch/medecine/antibodies/; University of Geneva, Switzerland).

Peptide-based C5 inhibitors (C5-inh; RA101495) and C3 inhibitors (C3-inh; compstatin, Cp20) were produced by microwave-assisted solid-phase peptide synthesis (CEM Liberty Blue; CEM GmbH, Kamp-Lintfort, Germany) using established protocols (29). The C5 inhibitor RA101495 was synthesized as derivative lacking the PEG24 and palmitoyl-substitution (present in the clinical compound to enhance *in vivo* circulation). The MASP-1 inhibitor SGMI-1 (MASP-1-inh) was produced as described previously (30, 31).

Recombinant fragments of FH, FH-like 1 (FHL-1) and miniFH were produced using a *Pichia pastoris* expression system as described previously (32–34). For a complete list, see **Supplementary Table 1**. In brief, coding sequences for the recombinant forms of FH were amplified from cDNA or a codon-optimized FH gene and cloned into the *P. pastoris* pPICZαB expression vector (Invitrogen). Recombinant proteins were secreted into the medium and purified by successive ion exchange and size exclusion chromatography steps.

The detection antibodies anti-C3-09 (35), anti-C3-19 (35), and anti-FH.16 (36) were fluorescently labeled in-house with DyLight 488 or 594 (Thermo Fisher Scientific, Allschwil, Switzerland). Anti-C3-09 and C3-19 recognize surface-deposited C3 activation fragments that contain the exposed C3c segment (*i.e.*, C3b, iC3b) or C3dg segment (*i.e.*, C3b, iC3b, C3dg), respectively. Anti-FH.16 recognizes an epitope in CCP16-17 of FH. Phalloidin 647 (1:400 diluted, Cat. Nr. UO298) was obtained from Thermo Fisher Scientific and DAPI (used at 1 µg/mL) from Sigma-Aldrich (Buchs, Switzerland).

### 2.3 Parasites

Cercariae of *S. mansoni* (Liberian strain) were obtained from infected intermediate host snails (*Biomphalaria glabrata*, Egyptian strain) and mechanically transformed to NTS as described previously (37). In short, *S. mansoni*-infected *B. glabrata* snails were placed singularly in 24-well plates and exposed to a neon lamp (36 W, 4000 K, 3350 lumen) for 3-4 h, to induce the shedding of cercariae. The supernatant was collected and filtered to remove impurities such as snail eggs from the suspension. Cercariae were then kept on ice for 30 min and centrifuged (3 min at 475 × *g*), before being mechanically transformed into NTS by physically removing the tail by constricted passage through a Luer-Lok tip in between two 12 mL syringes. NTS were sedimented by gravity at least 3 times for 7 min in ice-cold Hanks’ Balanced Salt Solution (HBSS containing 1.3 mM Ca^2+^ and 0.9 mM Mg^2+^; Gibco, Waltham MA, USA) supplemented with 1% (v/v) pen/strep, to separate them from the tails. The suspension was adjusted to 30 – 50 NTS per 50 µL HBSS, after which survival experiments were initiated immediately, unless otherwise indicated. For the preparation of 24 h-, 48 h-, and 72 h-old NTS, parasites were resuspended in NTS-medium and kept at 37°C and 5% CO_2_ until use. Adult *S. mansoni* of both sexes were collected from the hepatic portal system and mesenteric veins of mice, which had been infected with 100 cercariae 49 days earlier. The worms were washed with PBS supplemented with 1% (v/v) pen/strep solution and kept in RPMI 1640 culture medium supplemented with 1% (v/v) pen/strep solution and 5% (v/v) FCS at 37°C and 5% CO_2_ until use.

### 2.4 NTS survival assay – fresh NTS

Where indicated, freshly prepared NTS were pre-incubated for 2 h at 37°C in HBSS containing 0.5 mM physostigmine salicylate in Eppendorf tubes. NTS were centrifuged for < 30 s at 1500 rpm, after which the supernatant was removed and replaced with fresh HBSS. After three washing steps, NTS were transferred into the culture plate. Flat-bottom 96-well tissue culture plates were prepared with 100 µL NHS or HI-NHS. Complement inhibitors were added in a volume of 50 µL HBSS, before addition of 30 – 50 NTS in 50 µL HBSS. Recombinant FH fragments and miniFH were added at a final concentration of 4 μM. The NTS were then incubated for 24 h at 37°C and 5% CO_2_. Total numbers of NTS per well were counted using the 20x LD objective of a brightfield inverted Primovert or Axiovert 40 CFL microscope (Zeiss, Germany). Death was assessed by loss of membrane integrity (granular appearance) and lack of motility. The survival rate (%) was calculated by dividing the number of dead parasites on the total number of parasites per well. At least three independent experiments were performed, with each condition in duplicate. Images shown were obtained by using an Axiocam 105 color camera coupled to the Axiovert 40 CFL with ZEN Lite software.

### 2.5 NTS survival assay – 24h/48h/72h-old NTS

In an experimental procedure similar to the survival assay with freshly obtained NTS, 24 h-old NTS were challenged with NHS and PZQ for 72 h. PZQ and complement inhibitors were dissolved in NTS-medium and contained 0.1% DMSO to match the highest dose of PZQ at 10 µM. Survival was assessed at 24 h, 48 h and 72 h (*i.e.*, when NTS are 96 h old). Of note, NTS that were alive, but clearly affected by the presence of PZQ (and would have received a score of 0.5 or 1 in our *in vitro* drug screens (37)) were counted as alive. For indicated experiments, 48 h- and 72 h-old NTS were challenged with NHS and PZQ.

### 2.6 Microscopy

Parasites were prepared using similar conditions as for all survival assays, but incubated with NHS for 1 h in a 96-well flat-bottom plate. NTS were transferred to round-bottom plates to facilitate washing steps by centrifugation (1500 rpm for 2-3 min). After removal of 100 µL supernatant, 100 µL 7.4% formaldehyde was added to incubate for 15 min at RT while shaking (300 rpm on a Thermomixer (Eppendorf)), followed by three washing steps in PBS 2% (w/v) BSA and two washing steps in PBS. Each time, 100 µL supernatant was left in the wells, 150 µL buffer was added and plates were spun down by centrifugation. When preparing adult worms, incubation was done in 48-well flat bottom plates, with 1 or 2 pairs per well. Adult worms remained inside the wells while washing with volumes of 250 µL buffer. Staining of the parasites was done by incubating 45 min at RT with anti-C3-19^488^, anti-C3-09^488^, anti-FH.16^594^, phalloidin^647^ and/or DAPI in PBS 0.5% (w/v) BSA, as indicated. NTS were spun down, transferred in 10 µL volume to be mixed with 10 µL Vectashield Plus and mounted on Menzel Superfrost Plus slides (using coverslips thickness No. 1.5, Thermofisher Scientific). NTS were observed using a 40x objective (HCX PL Fluotar 40x/0.75 dry) and adult worms using a 20x objective (HC PL Fluotar 20x/0.50 dry) on a Leica DM5000B widefield fluorescence microscope using LAS X software. Image analysis was performed by Fiji (https://imagej.net), using automated thresholds based on the phalloidin fluorescence to create a selection that included the entire NTS. These selections were used to calculate mean fluorescence intensities (MFI).

### 2.7 Statistics

Prism (version 8; GraphPad Software, La Jolla, CA, USA) was used to analyze data and perform statistics. Significant differences were assessed by paired *t*-test or (repeated measurements) one-way ANOVA followed by *post-hoc* tests for multiple comparisons (Dunnett or Tukey) as indicated.

## 3 Results

### 3.1 NTS are vulnerable towards complement-mediated killing at the early stage of transformation

**Figure 1:**
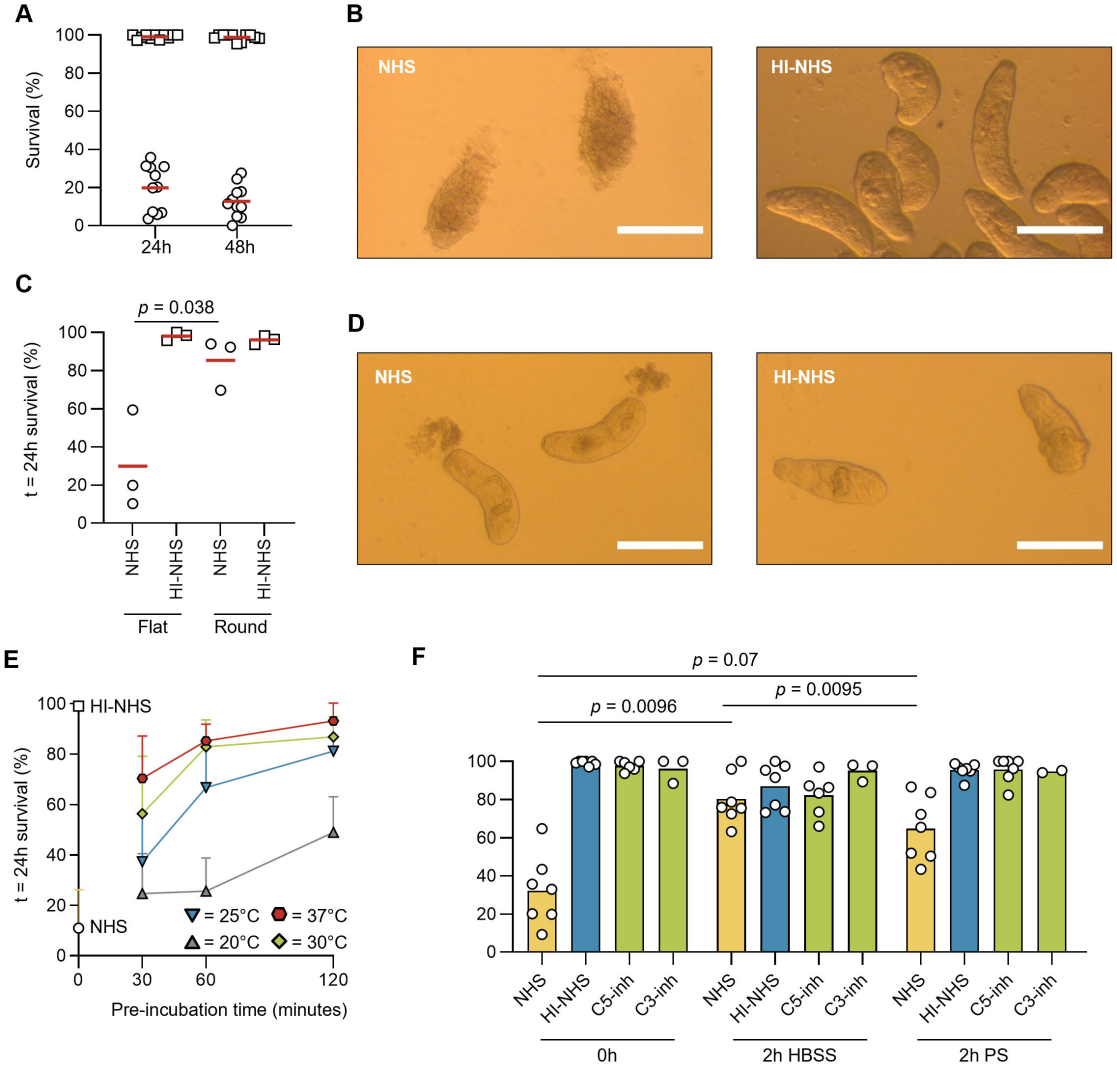
Complement resistance rapidly increases upon development of NTS. **(A)** Volumes of 100 µL HBSS containing 30 – 50 freshly prepared NTS were mixed with 100 µL heat-inactivated normal human serum (HI-NHS, *squares*) or NHS (*circles*, final concentration 50%) in flat-bottom 96-well culture plates and incubated at 37°C. Survival was assessed at t = 24h and t = 48h by counting total numbers of live and dead NTS on a brightfield microscope. Each symbol represents the mean of duplicate conditions, performed during independent experiments (*n* = 12). **(B)** Representative images of live and dead NTS after 24h incubation. Images were taken after 24h incubation using the 10x objective. Scale bars represent 1 mm. **(C)** Survival assay as in **(A)** using flat-bottom and round-bottom 96-well culture plates. Lines indicate mean, *n* = 3, paired *t*-test. **(D)** Representative images of NTS after 1h incubation in 50% serum, demonstrating acetabular gland release. **(E)** Freshly prepared NTS were pre-incubated for 30, 60 or 120 minutes in HBSS at increasing temperatures (20°C – 37°C), before being challenged with 50% NHS. Experiments (*n* = 4) were performed in duplicate. Survival was assessed as in **(A)**, lines indicate mean ± SD. **(F)** freshly prepared NTS were pre-incubated in HBSS or HBSS containing 0.5 mM physostigmine (PS) for 120 minutes, before being challenged with 50% NHS, HI-NHS, NHS containing C3-inh (Cp20) or C5-inh (RA101495) to block complement activation at the level of C3 or C5, respectively. Symbols indicate the mean of duplicate conditions, performed during independent experiments (*n* = 7). Bars indicate mean. RM one-way ANOVA followed by Tukey’s multiple comparisons test, on NHS (0h) vs NHS (2h HBSS) vs NHS (2h PS).

To analyze the vulnerability of invading *S. mansoni* towards complement-mediated killing, we used a setting in which freshly prepared NTS were only kept in cold physiological saline solution for limited time before the start of the experiments, in order to closely mimic the moment of skin penetration. When challenged with non-immune human serum, the majority of NTS did not survive when assessed after 24 h, and only minor additional killing was observed after 48 h (**Figure 1A**). The exposure to serum had a binary outcome, with NTS either being killed by complement-mediated damage or displaying a healthy phenotype similar to that after exposure to heat-inactivated serum. At the start of each experiment, NTS are dispersed around the well. In the control conditions, NTS would slowly gather in one corner of the well, with their tails or heads close to each other (**Figure 1B**). When being challenged with complement-active serum, however, the NTS lost the grouping phenotype and remained dispersed around the well, indicating that complement acted quickly before any directed movement could take place. Those NTS that survived the complement attack and displayed a healthy phenotype did not group in the remaining time of the experiments (72 h). When enforcing NTS grouping by use of a round-bottom plate, their survival increased profoundly (**Figure 1C**). We observed the rapid release of pre-formed secretory vesicles, the acetabular glands, within 1 h after NHS exposure (38, 39) (**Figure 1D**). These secretions contain proteases that will aid the NTS in migration through the skin. However, we hypothesize that these proteases will simultaneously inactivate complement components and thereby may provide an enhanced protective environment when grouped together (40, 41).

### 3.2 Resistance against complement-mediated killing increases with time and temperature

During penetration of the skin, parasites will need to adapt to a vastly different environment, featuring elevated temperatures and higher salt and protein levels. We therefore investigated the temperature- and time-dependence of NTS adaptation that results in developing resistance against complement-mediated killing. We incubated the freshly prepared NTS in HBSS at increasing temperatures, before addition of NHS. Even a short incubation of 30 min at 37°C already increased NTS survival to ~70%, with NTS achieving almost complete survival after 2 h (**Figure 1E**), which is in line with an early study by Marikovsky et al. (20). When incubated in the presence of physostigmine (PS), a reversible acetylcholinesterase inhibitor that halts cercarial transformation into NTS (25, 42), before being challenged with complement, NTS remained more vulnerable towards complement-mediated killing when compared to HBSS alone (65% vs. 80% survival after 24 h) (**Figure 1F**).

### 3.3 Complement-mediated killing is mainly caused by alternative pathway activation and terminal pathway effector functions

**Figure 2:**
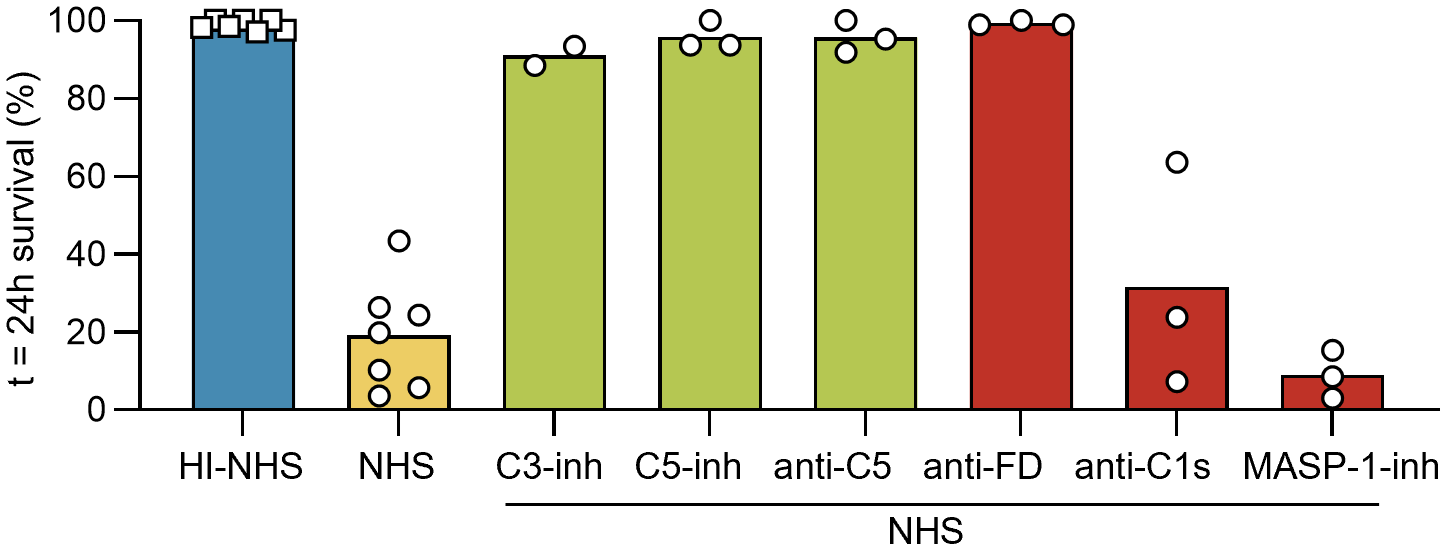
The alternative pathway is the main pathway involved in schistosomal killing in seronegative donor serum. Freshly prepared NTS were challenged with 50% NHS (or 50% HI-NHS as control) and incubated at 37°C. Survival was assessed at t = 24h by brightfield microscopy. Each symbol represents a single experiment performed in duplicate. Each condition was included in ≥ 3 independent experiments, except for C3-inh (*n* = 2). C5-inh and anti-C5 were added at 1 µM and 75 nM, respectively, to block the TP at the level of C5, while C3-inh was added at 10 µM to block at the level of C3. MASP-1-inh was used for the LP by blocking MASP-1 at 15 µM. The classical pathway was inhibited by use of anti-C1s at 100 nM and the alternative pathway was inhibited by anti-FD at 46 nM. Bars indicate mean.

In an attempt to identify the pathways responsible for the complement-mediated killing of NTS, we employed a panel of specific complement inhibitors. Compounds broadly blocking complement activation at the level of C3 [C3-inh; compstatin, Cp20 (43)] or C5 [C5-inh; zilucoplan surrogate (44) and anti-C5; eculizumab surrogate (45)] were able to rescue the NTS from killing. This indicates that the observed killing was indeed entirely complement-mediated and required the activation of the terminal pathway (**Figure 2**). When using initiation-pathway-specific inhibitors, only the impairment of the AP via an anti-factor D minibody [anti-FD; lampalizumab surrogate (46, 47)] had a substantial impact on survival, while no such effect was observed when blocking the CP using anti-C1s [sutimlimab surrogate (48)] or the LP using the MASP-1 inhibitor SGMI-1 (30, 31). This suggests that the AP is the main contributor to complement-mediated killing of NTS.

### 3.4 Surviving NTS remain under complement attack

**Figure 3:**
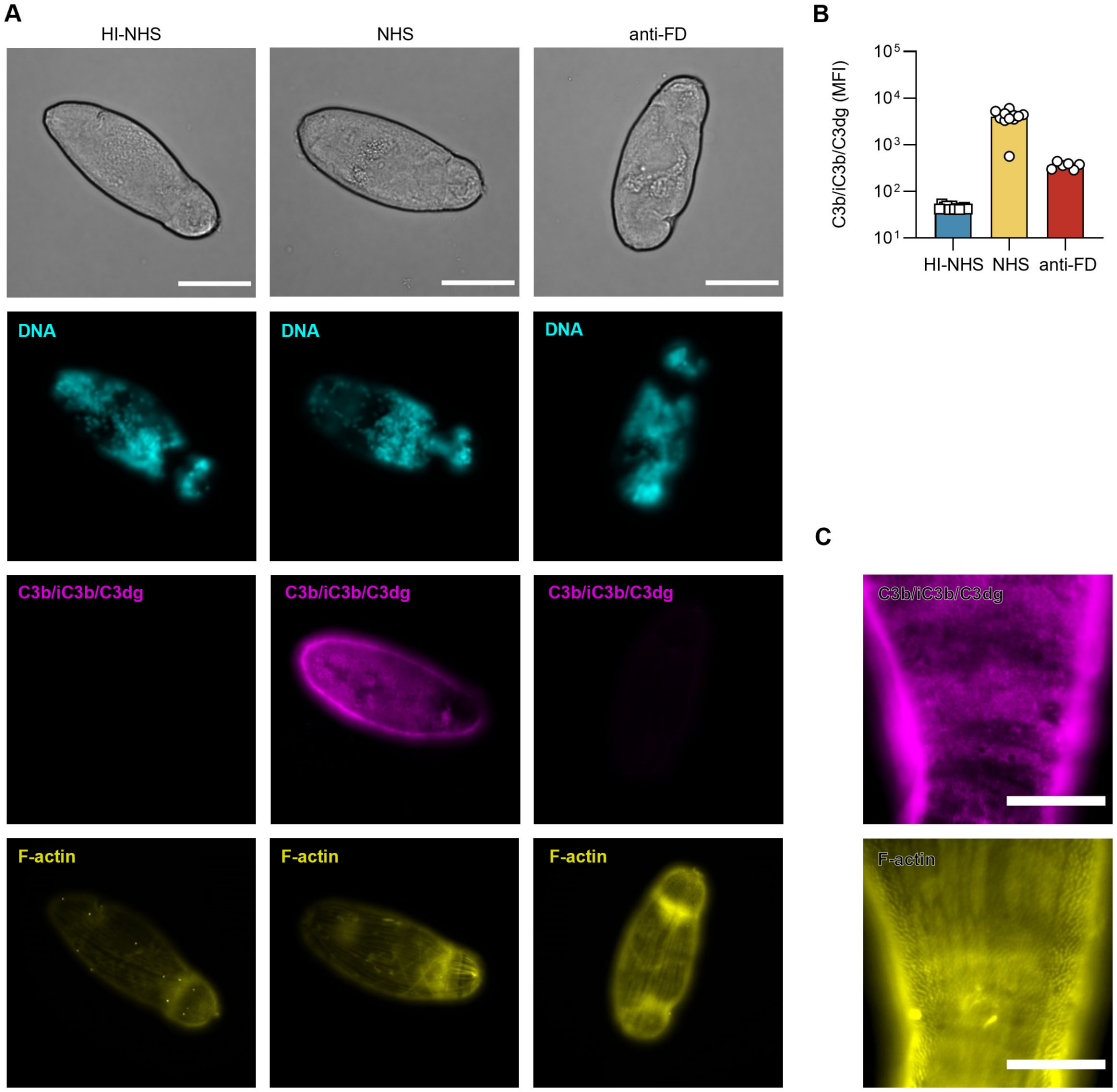
Surviving NTS remain under complement attack. **(A)** 2h-old NTS were challenged with 50% HI-NHS, NHS or NHS supplemented with 5 µg/mL anti-FD, and incubated at 37°C. After 1h, NTS were prepared for microscopy and stained for DNA (DAPI), F-actin (phalloidin^647^) and C3 fragments (C3b/iC3b/C3dg; anti-C3-19^488^). Images are from a representative experiment (*n* = 4). Scale bars indicate 50 µm. **(B)** Representative experiment (*n* = 4) depicting mean fluorescence intensity (MFI) for C3 fragments (C3b/iC3b/C3dg; anti-C3-19^488^) on a widefield fluorescence microscope using the 40x objective. Each symbol represents a single NTS (*n* = 6 for HI-NHS and anti-FD, *n* = 12 for NHS). Bars indicate mean. **(C)** Representative detailed images of an NTS showing deposition of C3 fragments on its surface, possibly located around actin-rich spines. Scale bar indicates 20 µm.

The observed resistance of NTS to complement-mediated damage could either be driven by a lack of recognition and opsonization via upstream activated elements of the cascade or by efficient prevention of MAC formation. We therefore stained surviving NTS for surface-bound C3 fragments (*i.e.*, C3b, iC3b, C3dg) and detected C3-derived opsonin deposition evenly distributed over the parasite tegument (*i.e.*, the outer surface) (**Figures 3A, B**). However, a more detailed look revealed many isolated dark areas, seemingly co-localizing with the actin-rich spines, that remained unstained (**Figure 3C**). Comparative staining with antibodies that either recognize C3c-containing (*i.e.*, C3b, iC3b) or C3d-containing opsonins (*i.e.*, C3b, iC3b, C3dg) resulted in identical staining patterns, which suggests that C3b either remains intact on the surface or is partially degraded to iC3b but not to the end-stage C3dg level (**Supplementary Figure 1**). This indicates that NTS remain prone to complement attack with resulting C3b deposition, but that this opsonization does not propagate into MAC-mediated killing.

### 3.5 Potential complement evasion by recruitment of factor H

**Figure 4:**
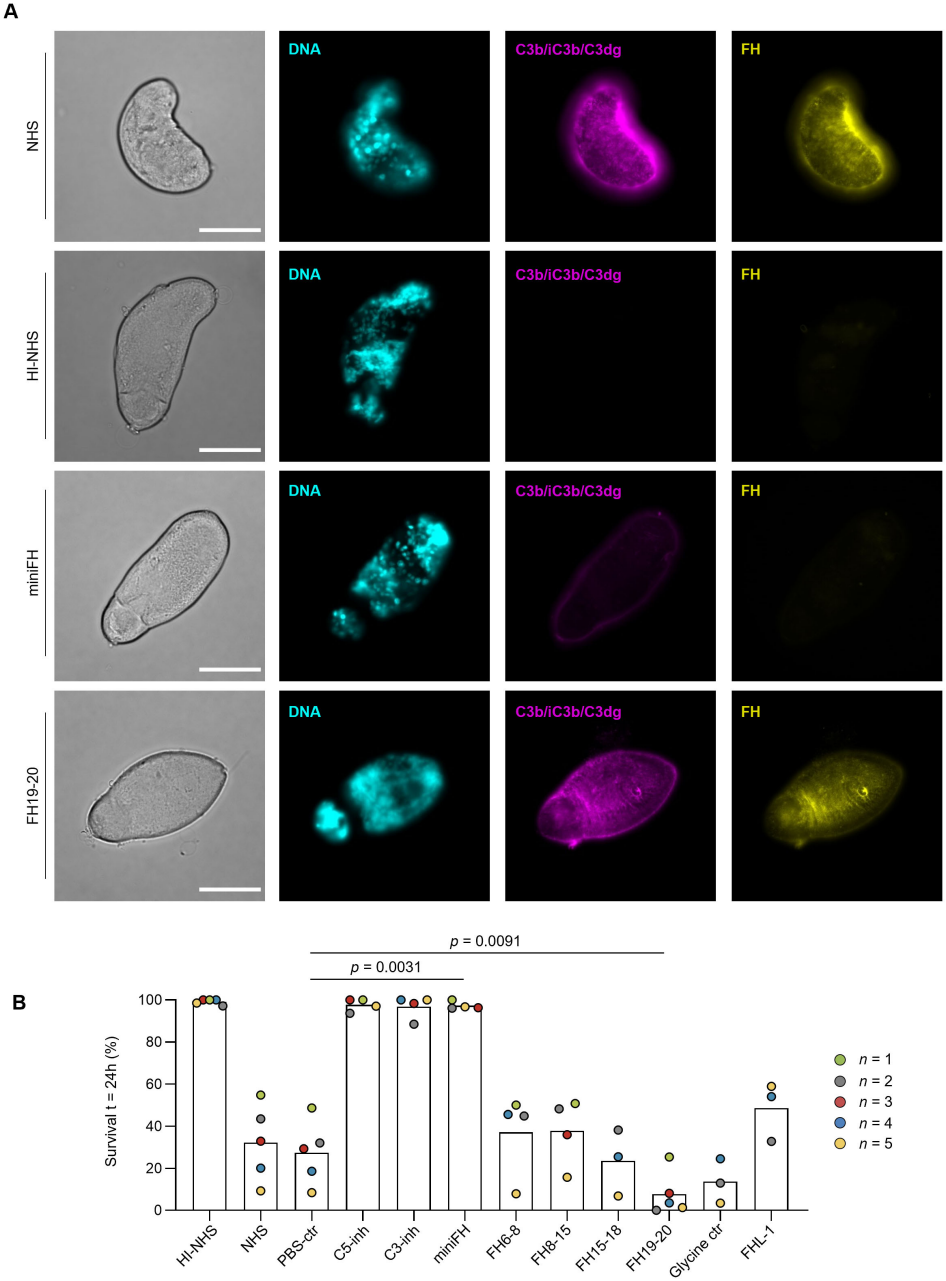
Binding of Factor H increases survival of freshly isolated NTS. **(A)** 24h-old NTS were challenged with 50% HI-NHS, NHS or NHS supplemented with 4 µM FH19-20 or 4 µM miniFH and incubated at 37°C. After 1h, NTS were prepared for microscopy and stained for DNA (DAPI), C3 fragments (C3b-iC3b/C3dg; anti-C3-19^488^) and FH (anti-FH.16^594^). As anti-FH.16 recognizes CCP16/17, it will only detect FH, and not miniFH. Scale bar indicates 20 µm. **(B)** Freshly isolated NTS were challenged with 50% NHS (or 50% HI-NHS as control) and incubated at 37°C. Survival was assessed at t = 24h by brightfield microscopy. Each symbol represents a single experiment performed in duplicate. Each condition was included in 3 – 5 independent experiments, as indicated in the legend. Control conditions included addition of C5-inh and C3-inh. Recombinant FH fragments and miniFH were added at 4 µM (dissolved in PBS). FHL-1 was added at 4 µM (dissolved in glycine buffer). Paired *t*-test on PBS-ctr vs miniFH or FH19-20.

The observation that NTS restrict the complement response to a level with limited effector function may suggest that host- or parasite-derived complement regulators contribute to the mechanism responsible for the rapid increase in complement resistance of freshly prepared NTS. While some pathogens produce distinct complement inhibitors, several microbial and parasitic organisms rather exploit the regulatory capacity of the host by recruiting plasma-derived regulators such as FH (49). Indeed, we could demonstrate that FH was bound to the schistosomal surface upon incubation of 24 h-old NTS with human serum (**Figure 4A**). We used the aFH.16 antibody, which specifically recognizes FH domains CCP16-17 and therefore only detects native FH but not recombinant FH fragments or miniFH that were added as modulators in this study. The C-terminal complement control protein domains (CCP19-20) of FH are often involved in cell surface binding, including those of microbial and parasitic targets (32, 49). Addition of a recombinant FH19-20 fragment as competitor led to a partial yet notable decrease in FH staining on the schistosomal surface, whereas the impact on opsonization was less obvious (**Figure 4A**, **Supplementary Figure 2**). In comparison, a much more marked drop in FH staining was observed when we added miniFH, a fusion construct between the regulatory and surface recognition domains of FH (CCP1-4/CCP19-20) (34). Notably, miniFH not only reduced FH staining but also impaired the deposition of C3 fragments to an almost undetectable level (**Figure 4A**). The stronger regulatory effect of miniFH over native FH may be explained by an enhanced selectivity towards early opsonins and an up to 10-fold reported activity improvement (34). Similarly, no FH or C3 fragments were detected when NTS were exposed to C3-depleted serum (**Supplementary Figure 3**).

To investigate the functional impact of FH recruitment, we measured the survival of freshly prepared NTS in serum containing miniFH or several short FH fragments. Addition of miniFH increased complement evasion and fully restored survival of the NTS to a level achieved by C3 and C5 inhibitors or observed in heat-inactivated serum (**Figure 4B**). The rescue effect of FH-like protein 1 (FHL-1), a short splice variant of FH that only contains CCP1-7, was notable yet much weaker when compared to miniFH. In contrast, FH6-8, FH8-15 and FH15-18 had little impact on survival whereas FH19-20 even led to a markedly decreased survival. These findings suggest that the C-terminal domains rather than central domains are involved in the recruitment of FH to the schistosomal surface, while the presence of the regulatory N-terminus of FH contributes to survival.

### 3.6 Praziquantel promotes vulnerability towards complement-mediated killing of NTS

**Figure 5:**
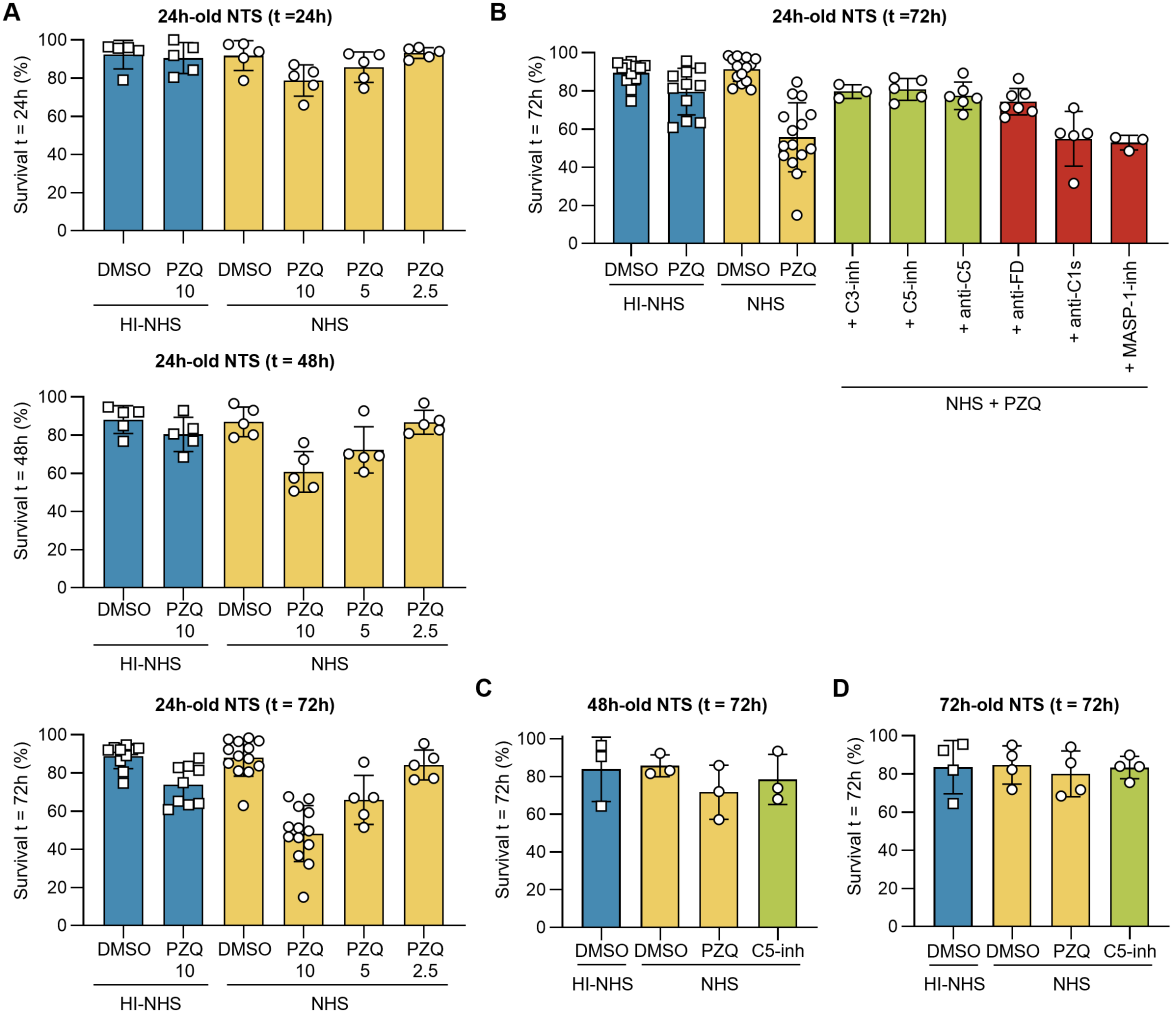
PZQ drives vulnerability towards complement-mediated killing. 24h-old NTS were challenged with 50% NHS and PZQ at 10, 5 or 2.5 µM, incubated at 37°C. Controls contained a DMSO concentration of 0.1%, matching the concentration of DMSO in the 10 µM PZQ conditions. **(A)** Survival was assessed after 24h, 48h and 72h by brightfield microscopy. Each symbol represents a single experiment (*n* ≥ 5) performed in duplicate. **(B)** Survival of 24h-old NTS after t = 72h in presence of 10 µM PZQ and complement inhibitors that act at the level of C3 (C3-inh), C5 (C5-inh, anti-C5), or pathway specific at factor D (AP, anti-FD), C1s (CP, anti-C1s) and MASP-1 (LP, MASP-1-inh). **(C, D)** Survival of 48h-old **(C)** and 72h-old **(D)** NTS in presence of 50% NHS and 10 µM PZQ. C5-inh was used at 1 µM to block TP activation. Each symbol represents a single experiment (**C**, *n* = 3; **D**, *n* = 4) performed in duplicate. Lines indicate mean ± SD.

Some schistocidal drugs, including PZQ, have been demonstrated to act in synergy with the immune system of the host [reviewed in (50)]. We therefore hypothesized that the surface-damaging effects of PZQ may enhance the vulnerability of NTS for complement-mediated killing (51). Indeed, when 24 h-old NTS were challenged with complement-active serum and PZQ simultaneously, we observed a reduced survival of the parasites depending on PZQ dose and incubation time, dropping to ~50% survival after 72 h in 10 µM PZQ (**Figure 5A**). Of note, the sensitizing effect was specific to PZQ as mefloquine (MFQ) and oxamniquine (OXA), two other drugs with antischistosomal properties (52–54), did not reduce survival of NTS (**Supplementary Figure 4**). We could confirm that the PZQ-enhanced killing was complement-mediated, since addition of either C3 inhibitors (C3-inh) or C5 inhibitors (C5-inh, anti-C5) rescued the NTS from killing (**Figure 5B**). In agreement with the pathway-dissection experiments described above, the AP-specific inhibitor (anti-FD) had a notable impact on the survival of 24 h-old NTS, whereas inhibition of the CP (anti-C1s) or the LP (MASP-1-inh) showed little to no effect. The synergy between PZQ and complement was lost when using 48 h- or 72 h-old NTS, suggesting that the mature parasites continue to develop the protective capacity of their outer surface (**Figures 5C, D**). Despite the PZQ-induced damage to the outer surface of NTS, the level of deposition of C3 fragments on the schistosomal surface was comparable to NTS that were not treated with PZQ (**Supplementary Figure 5**).

### 3.7 Adult worms get opsonized and show a distinct C3b deposition pattern

**Figure 6:**
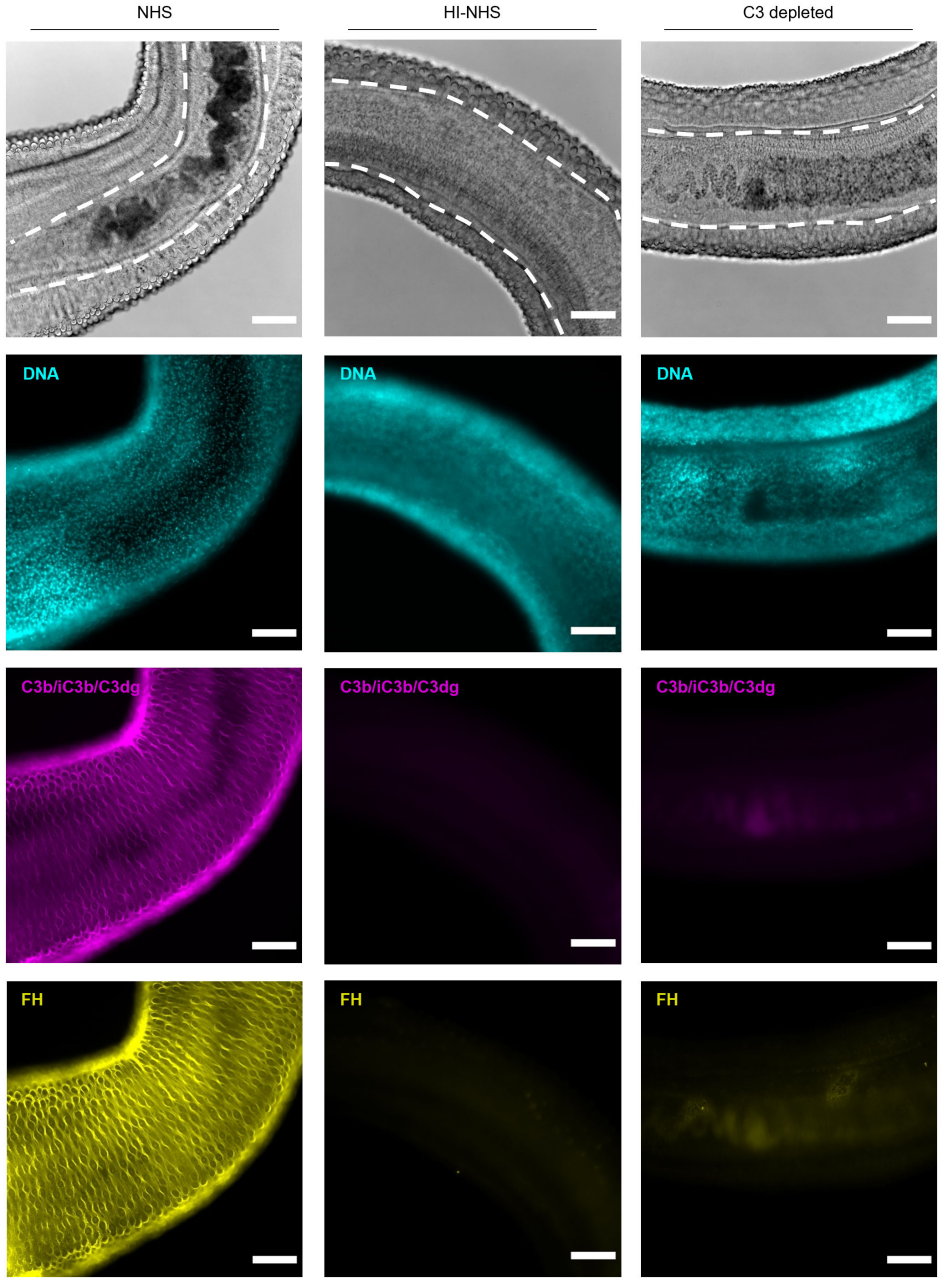
Adult worms remain under complement attack and recruit FH. Adult worms (mostly male-female pairs) were challenged with 50% HI-NHS, NHS or C3-depleted serum for 1h and incubated at 37°C. After 1h, adult worms were prepared for microscopy and stained for DNA (DAPI), C3 fragments (C3b/iC3b/C3dg; anti-C3-19^488^) and factor H (FH, anti-FH.16^594^). The main body parts of a male-female pair are depicted, with the female (within the dashed line in the top panels) residing in the ventral groove of the male being protected against complement attack. The digestive tract of the female worm is dark due to the presence of hemozoin, the result of blood digestion (top panels) and which also results in background fluorescence. Images are from a representative experiment (*n* = 6). Scale bar indicates 100 µm. Images are from a representative experiment (*n* = 6). Scale bar indicates 100 µm.

While it is established that adult worms can survive an attack by complement alone (23), our findings on NTS indicated that resistance to killing does not preclude that a complement response is triggered. Indeed, when challenging adult worm pairs with human complement, we observed deposition of C3 opsonins (**Figure 6**). As the female worm resides in the ventral groove of the male worm, they are protected against complement attack. Intriguingly, the outer surface of male worms showed an irregular staining pattern, where the ridges were profoundly opsonized (as measured by both anti-C3c and anti-C3dg antibodies, **Supplementary Figure 6**), whereas the tubercles in between the ridges remained unstained. A comparable staining pattern was observed for FH, again indicating that opsonization and regulation are influenced by each other. Although some degree of passive adsorption of complement proteins cannot be fully excluded, the observation that C3 deposition does not occur in HI-serum and FH cannot be detected when exposing worms to C3-depleted serum (**Supplementary Figure 6**) support the hypothesis that a functional connection between complement activator and regulator acquisition also applies to adult worms, albeit with less direct consequences on survival.

## 4 Discussion

In this study, we investigated the vulnerability of *S. mansoni* schistosomula towards complement-mediated destruction in relation to the stage of the parasite, the contribution of distinct complement pathways and regulators, and the presence of sensitizing schistocidal drugs. We could demonstrate that the susceptibility of freshly prepared NTS towards complement-mediated killing is short-lived, with NTS quickly gaining resistance when grouped or incubated in warm buffer before the serum challenge. The highly transient and context-dependent complement vulnerability of NTS observed in our experiments may also explain why earlier studies often differed in assessing the killing potential of complement (18–28), as such evaluations may strongly depend on the preparation of the parasites and on assay conditions. Some of the freshly prepared NTS are able to survive the challenge with complement-active serum and appear as viable as their counterparts exposed to heat-inactivated serum. When considering that the known transformation inhibitor physostigmine had a negative impact on the fraction of surviving NTS, it appears likely that some NTS have progressed more rapidly in ramping up their evasion strategies than others.

Importantly, surviving schistosomes, and even mature NTS and adult worms that are generally considered ‘complement-resistant’, remain under constant complement attack despite not being directly killed by MAC. While the immunological and functional implications of the profound opsonization detected on schistosomal surfaces remains to be explored, the presence of C3 fragments in their functionally active forms (C3b and/or iC3b) would imply that ligation by complement receptors (CRs) on phagocytes and other immune cells may occur. As it has been demonstrated for other pathogens (55), deposited C3b could be bound by CR1 on erythrocytes to result in the coverage of the entire parasite by erythrocytes as means to shield or shuttle the intruders. At the same time, the interaction of iC3b (and partially C3b or C3dg) with the integrin receptors CR3 and CR4 on phagocytes and with CR2 on B lymphocytes may at least theoretically induce downstream immune responses (11). Whereas all C3-derived opsonins may engage with CRs and modulate immune profiles, only intact C3b is capable of forming new C3 convertases and thereby amplify opsonization via the AP. On host cells, both the amount and degradation stage of C3 fragment deposits is strictly controlled by complement regulators on the cell membrane or, as in the case of FH and FHL-1, in circulation (56).

Given the impact of C3-derived opsonization on the fate of intruders, it is not surprising that many pathogens exploit FH and other regulators as part of their immune evasion strategy (55, 57, 58). Active recruitment of FH is not only employed by bacteria and viruses but also by protozoa and other parasites (57, 58). Our observation that serum-exposed NTS bind FH on their surface therefore appears to add *Schistosoma* to the growing list of FH-recruiting organisms. Despite being a common evasion mechanism, the exact binding mode and functional implications may vary considerably. On host cells, FH binding typically occurs by recognition of self-signatures via domains in the core (CCP7) or C-terminus (CCP19-20) of the regulator, whereas the N-terminus (CCP1-4) is responsible for destabilizing convertases and degrading C3b (56). Our experiments did not conclusively reveal how FH is recruited to schistosomal surfaces. While some pathogens express unique FH-binding entities (49), such a capturing molecule has not yet been described for *S. mansoni*. Yet, it cannot be excluded that this parasite engages a distinct mechanism or that FH binding is a consequence rather than a mode of evasion processes. Typically, the active recruitment of FH would provide a means to prevent high-density opsonization, with a negative correlation between FH and C3 fragment levels. In our case, the reduction of opsonization led to a decrease of FH binding and vice versa. This could indicate that surface-bound FH is determined by the presence of intact C3b at high densities rather than the expression of a parasite-derived FH-recruiting entity. Competition experiments with a panel of recombinant FH fragments covering the various functional segments of the regulator indeed indicate that fragments, which contain C3b-interacting domains (*i.e.*, CCP1-4, CCP19-20) have a bigger impact on binding and survival than the core sections. It has to be noted, however, that the C-terminal domains are shared with the family of FH-related proteins (FHR’s), which have been shown to exert counterregulatory effects in a surface-dependent manner (59). Future studies will need to further delineate whether and how opsonin-mediated binding, active recruitment and/or counterregulation contribute to the FH interaction profile of *S. mansoni*. In the same context, one should investigate the peculiar opsonization and FH-binding pattern on the adult worm tegument, with clear difference between ridges and intermittent spaces, to elucidate which surface elements are responsible for this distinction.

The high opsonin densities with concurrent FH binding in absence of MAC-mediated killing, at least in mature NTS and adult worms, also raises the question how the parasite achieves resistance. The difference in complement vulnerability between freshly prepared NTS and later states appears to rely both on how quickly complement activity can propagate to the terminal pathway, as indicated by the impact of the optimized FH construct miniFH on the survival of fresh NTS, and on the formation and damaging effect of MAC. Although we were unable to visualize any deposition of C5b or the MAC to confirm whether complement activation on surviving parasites goes beyond the level of C3, it is likely that the high C3b densities at least lead to the formation of C5 convertases. The lack of MAC efficiency could rather be determined by the surface structure that hampers the insertion of (sublytic) MAC and/or pore formation, or the presence of parasite-derived MAC inhibitors.

At least in the serum of non-exposed donors, which are expected to be seronegative for anti-schistosomal antibodies, the complement-mediated killing observed in our study was found to be mainly driven by the alternative pathway of complement activation. Among pathway-specific inhibitors, only a minibody that blocks the C3 convertase-forming protease factor D and reflects the activity of the clinically developed AP inhibitor lampalizumab notably affected survival rates. What remains to be determined is how the AP would be triggered by the schistosomal surface. In the case of other pathogens, surface-exposed entities such as lipopolysaccharides were identified as direct inducers of the AP (60). At the same time, the strong co-dependence of C3b deposition and FH binding could indicate that the recruitment of FH and/or FHR’s may have a unique or even counterintuitive effect on opsonization and AP activation. Although a direct recruiting of FH to the schistosomal surface, similar to evasion mechanisms observed in many pathogens (49), cannot be excluded, our data rather suggest that FH binding may largely depend on prior opsonization with C3 fragments. Among the supportive experiments for this hypothesis is the competition between FH and derived fragments for surface binding. FH itself has two opsonin interaction sites, though the cryptic nature of one of those regions appears to define the relative selectivity of the regulator for the degradation fragments iC3b and C3dg. Whereas miniFH, an engineered, truncated FH derivative designed for therapeutic applications, shares the oposonin binding sites with the parental regulator, its smaller size and improved accessibility of both contact regions was shown to strongly enhance C3b selectivity and regulatory activity (61). FH19-20, on the other hand, corresponds to the C-terminal recognition domains of FH and only contains a single opsonin binding site. The observation that miniFH strongly and FH19-20 weakly competes with schistosomal FH recruitment favors a prominent role of C3b-driven recruitment of FH. It has to be noted that FH19-20 competition by itself is not a conclusive indicator in our assay since some microorganisms and parasites do recruit FH via this fragment, but a more dominant role of this effect would have been expected in such a case. Another limitation is that the available detection antibodies and the kinetic restraints of the assays do not allow for a clear distinction between the presence of C3b or its degradation fragments and, hence, whether the recruitment of FH affects its regulatory capacity. While additional experiments are certainly warranted, our study provides a strong rationale for an evasion mechanism based on tandem binding of opsonins and regulators.

Although an involvement of the LP was previously suggested by the presence of MBL on the schistosomal surface, our studies could not confirm a notable contribution of this pathway on complement-mediated effector functions. We were unable to visualize any deposition of C4b (data not shown). In contrast, and as suggested previously (19), it is highly plausible that CP activation will be involved in the outcome if serum from schistosomiasis patients is used, due to the presence of anti-schistosomal antibodies. In future studies, it will therefore be important to further dissect how antibody-mediated CP activation, induction and amplification via the AP, opsonization, and regulator recruitment impact each other when defining susceptibility and resistance of the parasite to complement-induced damage.

Among the most intriguing findings of our study was the interplay between PZQ and complement in the killing of NTS. Most of the compounds employed for the treatment of schistosomiasis were shown to impact the integrity of the schistosomal surface (51, 62–64). One could therefore assume that, if relevant at all, any drug-induced tegumental damage would increase complement-mediated killing of NTS. However, in our experiments, such a complement-sensitizing effect was only evident in presence of PZQ and not in the case of MFQ or OXA. The addition of complement inhibitors confirmed that the drop of survival was facilitated by PZQ yet clearly mediated by complement effector functions. Moreover, the mode of complement engagement did not seem to be notably impacted by the drug, as the AP remained the dominant contributor to complement-mediated killing. Subsequent studies about how different antischistosomal drugs alter the cell surface on a molecular level could also help identifying the surface entities that are responsible for the activation of the AP and/or binding of FH. Beyond this mechanistic level, this and subsequent studies may open new avenues towards more effective or extended use of existing drugs such as PZQ or novel concepts that efficiently engage complement-sensitizing and -engaging modes of action across several schistosome lifecycle stages.

## Data Availability

The raw data supporting the conclusions of this article will be made available by the authors, without undue reservation.
