# Supplementary material for: Praziquantel and factor H recruitment differentially affect the susceptibility of Schistosoma mansoni to complement-mediated damage
Source: Front Immunol. 2024 Nov 12;15:1474358. doi: 10.3389/fimmu.2024.1474358 (PMC11588701; doi:10.3389/fimmu.2024.1474358)
Supplement: Supplementary file 1 [file DataSheet1.docx]

Supplementary Material

**Supplementary Table 1. Recombinant FH constructs.**

| **Recombinant FH constructs:** | **Recognized by anti-FH.16** | **Containing CCPs:** | **Buffer:** |
| --- | --- | --- | --- |
| FH6-8 | No | 6-8 | PBS |
| FH8-15 | No | 8-15 | PBS |
| FH15-18 | Yes | 15-18 | PBS |
| FH19-20 | No | 19-20 | PBS |
| FHL-1 | No | 1-7 | PBS |
| miniFH | No | 1-4 & 19-20 | 20 mM glycine, 150 mM NaCl pH 10.5 |

*CCP: complement control protein domain.*


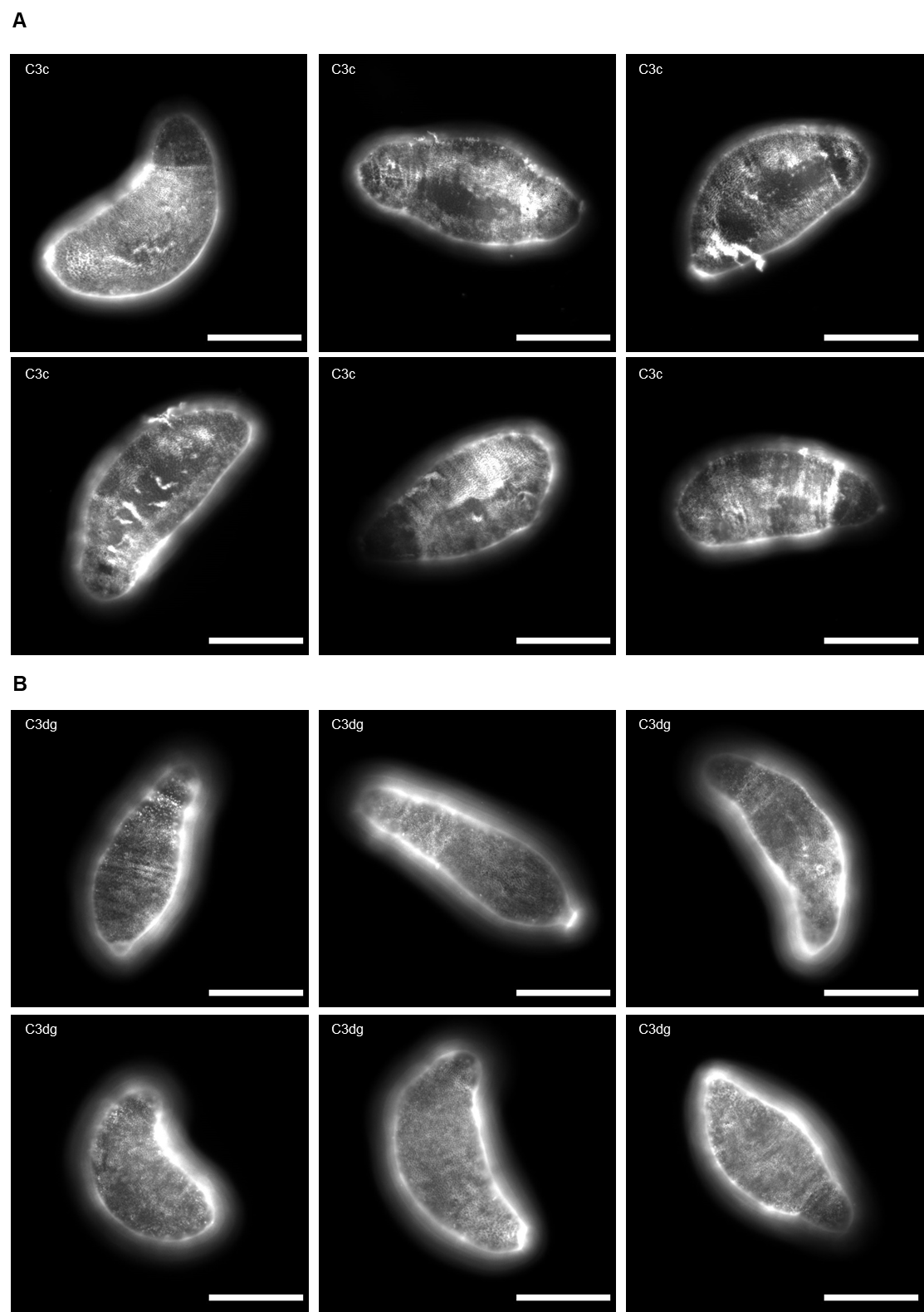


**Supplementary Figure 1. Similar patterns observed when staining for C3c and C3dg on NTS. (A)** Additional detailed images of NTS showing C3c deposition on its surface (anti-C3-09^488^), possibly located around actin-rich spines. Disturbed staining is presumably due to damage of the tegument during the fixation and washing steps. **(B)** Similar to **(A)**, deposition of C3dg (anti-C3-19^488^). Images were manually adjusted for brightness/contrast to maximize visibility of staining pattern. Scale bars indicate 50 µm.


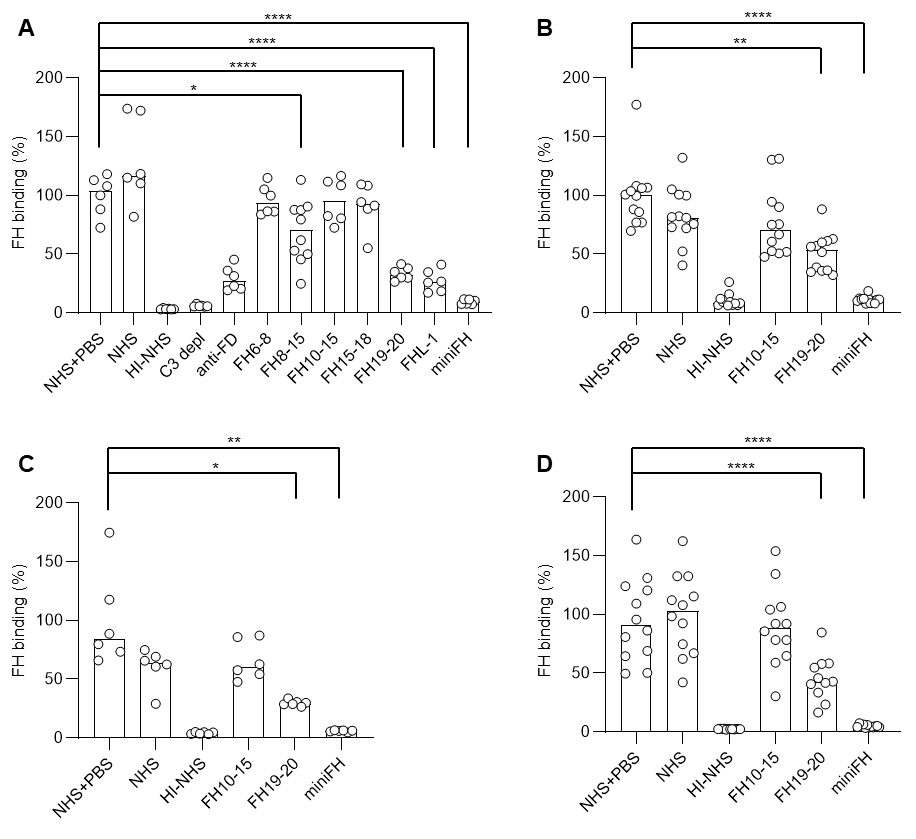


**Supplementary** **Figure 2. Addition of a recombinant FH19-20 fragment decreases FH staining on schistosomal surface.** 24h-old NTS were challenged with 50% HI-NHS, NHS or NHS supplemented with 4 µM of a recombinant FH fragment (FH6-8, FH8-15, FH10-15, FH15-18 or FH19-20), 4µM FHL-1 or 4 µM miniFH, or an equivalent volume of PBS and incubated at 37°C. Additional controls included C3-depleted serum and NHS treated with anti-FD. After 1h, NTS were prepared for microscopy and stained for FH (anti-FH.16^594^). Each panel represents an experiment, with each symbol representing a single NTS. Bars indicate median. FH binding in presence of FH19-20 is 50-70% lower than in NHS+PBS. One-way ANOVA, followed by Dunnett’s multiple comparisons test. * = *p* < 0.05, ** = *p* < 0.01, **** = *p* < 0.0001.


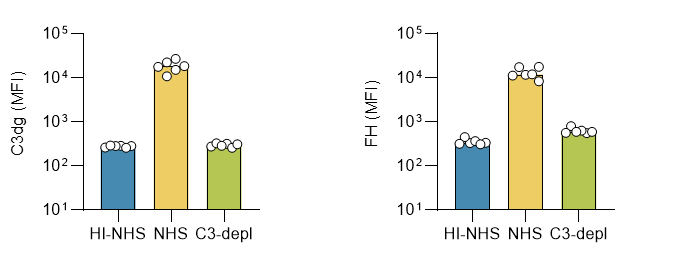


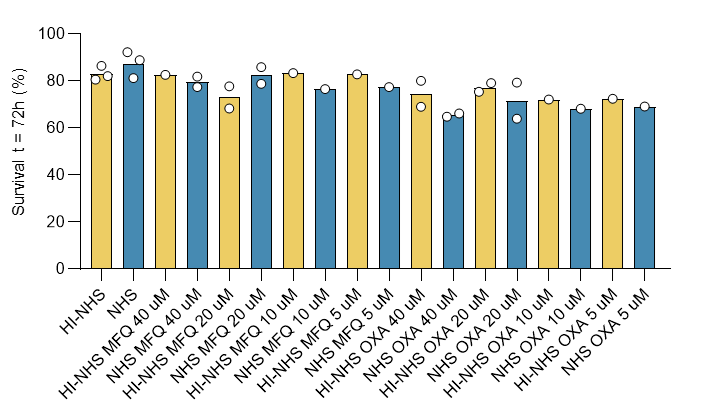
**Supplementary Figure 3. Binding of Factor H appears dependent on presence of C3 fragments.** 24h-old NTS were challenged with 50% HI-NHS, NHS or C3-depleted serum (C3-depl) and incubated at 37°C. After 1h, NTS were prepared for microscopy and stained for DNA (DAPI), F-actin (phalloidin^647^) C3dg (anti-C3-19^488^) and FH (anti-FH.16^594^). Each symbol represents a single NTS (*n* = 6). Bars indicate mean.

**Supplementary Figure 4. Mefloquine (MFQ) and Oxamniquine (OXA) do not drive vulnerability towards complement-mediated killing.** 24h-old NTS were challenged with 50% NHS and MFQ or OXA at 40, 20, 10 or 5 µM, incubated at 37°C. Controls (HI-NHS indicated in yellow, NHS indicated in blue) contained a DMSO concentration of 0.1%, matching the concentration of DMSO in the highest MFQ/OXA conditions. Survival was assessed after 72h by brightfield microscopy. Each symbol represents a single experiment performed in duplicate.


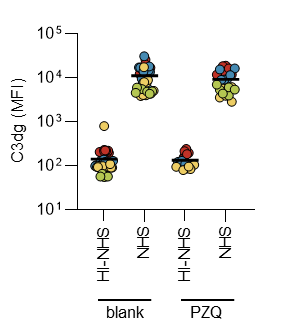
**Supplementary Figure 5. Equal levels of C3 fragments found on NTS challenged with complement in presence of PZQ.** 24h-old NTS were challenged with 50% NHS and 10 µM PZQ, incubated at 37°C. After 1h, NTS were prepared for microscopy and stained for DNA (DAPI), F-actin (phalloidin^647^) and C3dg (anti-C3-19^488^). Each symbol represents an NTS (n ≥ 6), each color a separate experiment (*n* = 4), lines indicate mean. At least 6 NTS per condition were assessed for their MFI.


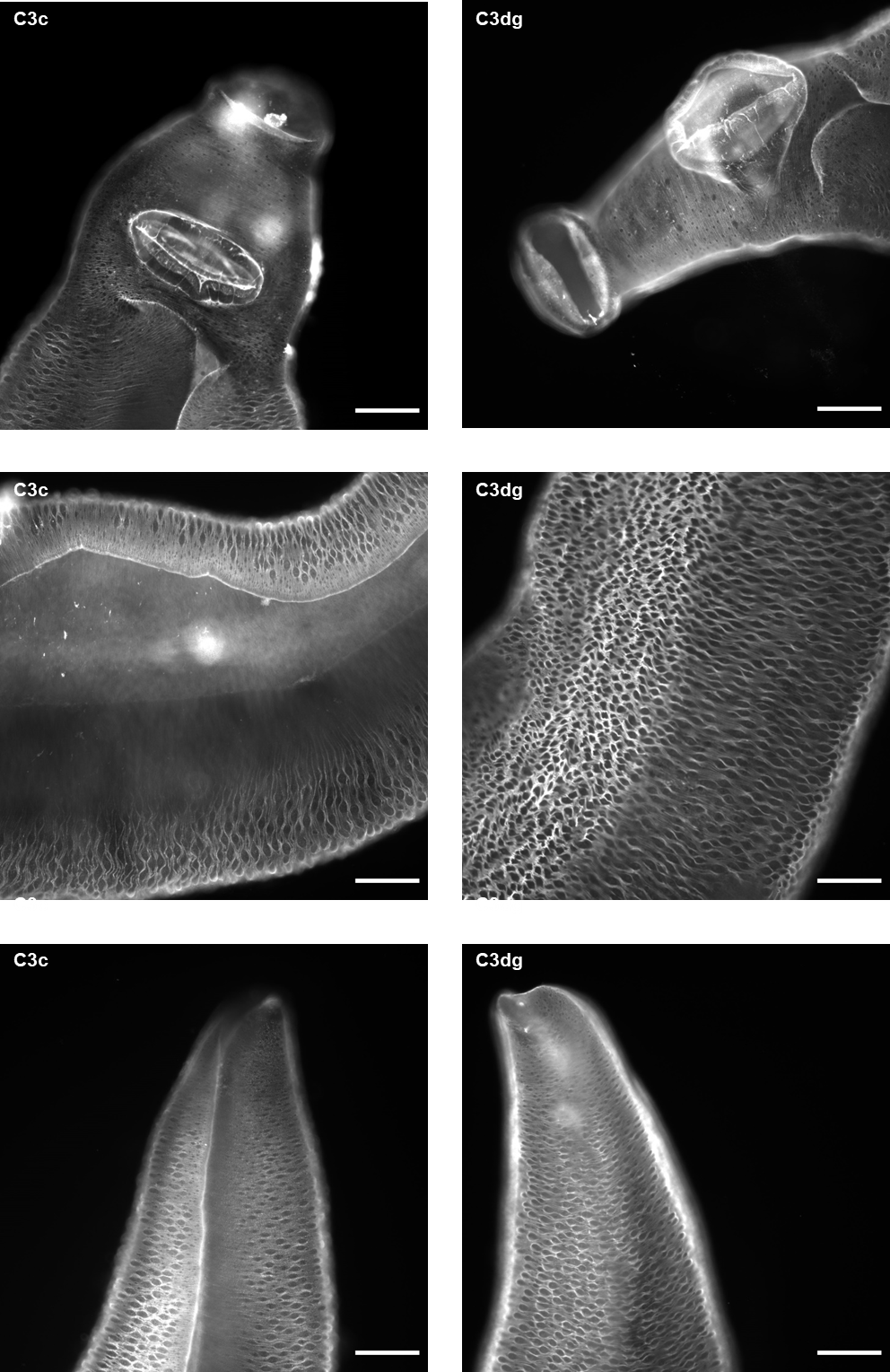


**Supplementary Figure 6. Similar patterns observed when staining for C3c and C3dg on adult male worms.** Images of adult male worms showing deposition of C3c (anti-C3-09^488^) or C3dg (anti-C3-19^488^) fragments. Upper panels depict male heads with the oral and ventral suckers; middle panels the main body; lower panels the tail ends. Images were adjusted for brightness/contract to maximize visibility of the staining pattern. Scale bars indicate 100 µm.
